# SEX AND RACE/ETHNIC DIFFERENCES IN TERMINAL COGNITIVE DECLINE

**DOI:** 10.1093/geroni/igad104.3095

**Published:** 2023-12-21

**Authors:** Iván Mejía-Guevara

**Affiliations:** Stanford University, Stanford, California, United States

## Abstract

**Background:**

Cognitive decline has been documented in older adults, but sex and racial/ethnic differences among decedents have not been studied. Here we investigate the relation of mortality to change in the cognitive function of older individuals by sex and race/ethnicity.

**Methods:**

The study analyzed data from the Health and Retirement Study (HRS) between 1993 and 2018, which included information on total word recall, mental status, and total cognition of individuals aged 65 and older. The study estimated the loss of cognitive function using a multilevel random coefficient model, considering sex and racial/ethnic differences in the risk of death, age, and death status. The study also approximated terminal cognitive decline using cognitive scores reported within two years before death for decedents.

**Results:**

On average, older women performed better than older men in word recall and overall cognition, while older White Americans performed better than African Americans and Hispanics/Latinos in overall cognition at all ages. Decedents had a similar cognitive function to survivors at age 65, but their cognition declined quickly with age, especially among older women and White Americans. Evidence of terminal cognitive decline within two years before death was significant regardless of age but less conclusive for African Americans and Hispanics/Latinos due to smaller sample sizes.

**Conclusions:**

This paper indicated evidence of sex and race/ethnic differences in cognitive decline and terminal cognitive decline among older adults, which may have critical clinical implications directly linked to the onset and duration of dementia risk.

